# Evaluation of Oral Cavity DNA Extraction Methods on Bacterial and Fungal Microbiota

**DOI:** 10.1038/s41598-018-38049-6

**Published:** 2019-02-06

**Authors:** Jennifer Rosenbaum, Mykhaylo Usyk, Zigui Chen, Christine P. Zolnik, Heidi E. Jones, Levi Waldron, Jennifer B. Dowd, Lorna E. Thorpe, Robert D. Burk

**Affiliations:** 1 Department of Pediatrics, Albert Einstein College of Medicine, Bronx, NY USA; 2 Department of Microbiology, Faculty of Medicine, The Chinese University of Hong Kong, Hong Kong, SAR China; 3 Department of Biology, Long Island University, Brooklyn, NY USA; 4 CUNY Graduate School of Public Health and Health Policy, New York, NY USA; 5 Departments of Obstetrics & Gynecology and Women’s Health, Epidemiology and Population Health, and Microbiology & Immunology, Albert Einstein College of Medicine, Bronx, NY USA; 6 Department of Global Health and Social Medicine, King’s College London, London, UK; 7 NYU School of Medicine, Department of Population Health, New York, NY USA

## Abstract

The objective of this study was to evaluate the most effective method of DNA extraction of oral mouthwash samples for use in microbiome studies that utilize next generation sequencing (NGS). Eight enzymatic and mechanical DNA extraction methods were tested. Extracted DNA was amplified using barcoded primers targeting the V6 variable region of the bacterial 16S rRNA gene and the ITS1 region of the fungal ribosomal gene cluster and sequenced using the Illumina NGS platform. Sequenced reads were analyzed using QIIME and R. The eight methods yielded significantly different quantities of DNA (p < 0.001), with the phenol-chloroform extraction method producing the highest total yield. There were no significant differences in observed bacterial or fungal Shannon diversity (p = 0.64, p = 0.93 respectively) by extraction method. Bray-Curtis beta-diversity did not demonstrate statistically significant differences between the eight extraction methods based on bacterial (R^2^ = 0.086, p = 1.00) and fungal (R^2^ = 0.039, p = 1.00) assays. No differences were seen between methods with or without bead-beating. These data indicate that choice of DNA extraction method affect total DNA recovery without significantly affecting the observed microbiome.

## Introduction

The human oral cavity hosts a diverse microbial community comprised of bacteria, fungi, protozoa, archaea, and viruses^1^. The vast bacterial biota includes pathogenic bacteria that are responsible for local and systemic diseases^2^. For example bacteria have been shown to be responsible for oral ailments such as dental caries^3^, and periodontal diseases^4^. The scope of bacteria causing oral ailments is also vast with conditions such as mild gum disease and gingivitis affecting over 90% of adults^5^ at some point in their lives. Oral bacteria may also be related to diseases not localized to the oral cavity, such as diabetes^6^, cardiovascular disease^7^, chronic respiratory conditions^8^, rheumatoid arthritis^9^, malignancy^10,11,13,14^, preterm labor and low birth weight^12^. In addition to bacteria, the oral cavity hosts a variety of fungal species^15^. Despite this, the fungal constituents of the oral microbiome have thus far been understudied when compared to bacteria, but are now emerging as being important in human disease. For example, fungi have been recently shown to affect treatment outcomes in immunocompromised individuals^16^ as well as the development of colorectal cancer^17^. Moreover, studies also indicate that fungi operate together with bacteria in oral infections^18^.

Since the oral cavity is a potential reservoir for organisms implicated in oral and systemic health, it is essential to determine the appropriate molecular assays to study its entire microbiome including the fungal communities. Initially, studies of the oral microbiome focused exclusively on pathogenic organisms and utilized culture-based techniques. However, with the knowledge that the oral microbiome is dominated by non-culturable species^19^, use of culture-independent molecular methods has increased. One of the most commonly used techniques involves high-throughput, massively parallel amplicon-based sequencing and subsequent taxonomic assignment based on publicly available reference databases^20^. The characterization of the microbial communities using this platform can be influenced at several steps including sample collection, DNA extraction, PCR amplification, sequencing, data processing, and statistical analyses^21^. Additionally, each of these steps has associated labor and cost factors that may influence a researcher’s decision to use one method over another^22^. Previous research has shown that oral sampling techniques such as saliva, buccal swab, and oral rinse collection may influence overall DNA quantity and spectrum of microbes detected^23–25^. It has also been suggested that next-generation sequencing (NGS) may produce variable results particularly when analyzed using different classification algorithms^26^. Given that these processes can influence the understanding of microbial communities, investigating protocols for characterizing the biota of the oral cavity is important to allow inter-study comparisons.

Efficient and consistent methods of DNA extraction are central to accurately characterizing these communities. A number of studies have begun to examine the oral microorganisms using NGS with a variety of DNA extraction methods^27–33^. In addition, a large number of studies have collected and processed Scope mouthwash samples for genomic DNA that might be suitable for microbiome studies. The purpose of this investigation was to compare the most recent techniques to discern the most effective method of DNA extraction utilizing both enzymatic and mechanical lysis techniques across various human oral samples in order to determine the methods with the highest DNA yield and the most consistent results for characterization of both bacterial and fungal communities found in the oral cavity.

## Results

**Figure 1:**
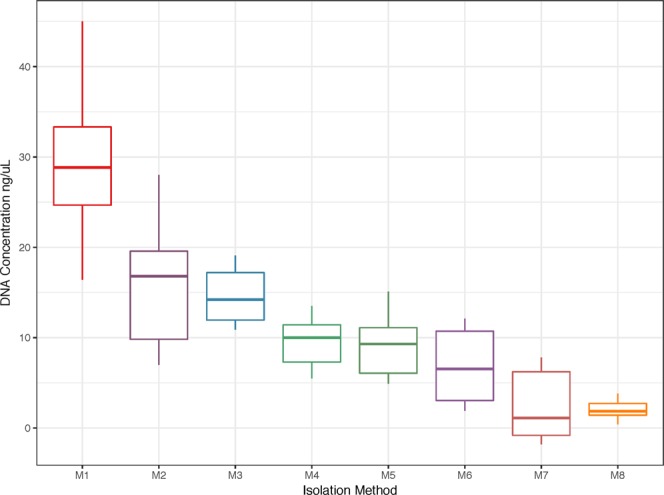
DNA Quantitation for each isolation method. DNA concentrations (ng/μl) of six oral samples were calculated after eight different DNA extraction methods described in Table 1 and corresponding to the categories shown on the x-axis. All methods used the same starting quantities of sample and final volumes were equal; concentrations are proportional to total DNA recovered. Statistical analyses of the differences in DNA amounts recovered are shown in Table 2.

For this study, eight DNA extraction methods, utilizing different combinations of enzymatic and mechanical lysis techniques, were compared across six oral samples (Table 1). The methods were evaluated for DNA yield and variation in the detected oral microbiome. There was a significant difference in DNA quantity among the eight extraction methods (p < 0.001). The phenol-chloroform extraction technique (Method 1) generated the highest DNA yield (Fig. 1) while the UltraClean Microbial DNA Isolation Kit (Method 7) and the UltraClean Microbial DNA Isolation Kit (Method 8) resulted in significantly lower DNA yields (p < 0.01) than the three non-bead-beating methods (Table 2).

**Table 1:** Methods of DNA extraction used in this study, including additional enzymatic and mechanical (bead-beating) cell disruption steps.

Method	Extraction Method	Commercially Available Kit	Enzymatic Lysis Step Added	Bead-beating
M1	Phenol/chloroform	No	No	No
M2	QIAamp DNA Mini Kit	Yes	No	No
M3	QIAamp DNA Mini Kit		Mutanolysin	No
		Yes	Lysozyme	
			Lysostaphin	
M4	QIAamp DNA Mini Kit		Mutanolysin	0.1 mm-diameter zirconia/silica beads (BioSpec)
		Yes	Lysozyme	
			Lysostaphin	
M5	PowerLyzer PowerSoil DNA Isolation Kit		Mutanolysin	0.1 mm-diameter glass beads (MoBio)
		Yes	Lysozyme	
			Lysostaphin	
M6	PowerSoil DNA Isolation Kit	Yes	No	0.7 mm-diameter garnet (MoBio)
M7	UltraClean Microbial DNA Isolation Kit	Yes	No	0.7 mm-diameter garnet (MoBio)
M8	BiOstic Bacteremia DNA Isolation Kit	Yes	No	0.15 mm-diameter garnet (MoBio)

Enzymes listed in this table are in addition to any lysis buffer included in each kit (either specified, such as proteinase K, or proprietary). All bead-beating was conducted on a FastPrep-24 Instrument (MP Biomedicals) at 6.0 m/s for 40 seconds.

**Table 2:** Tukey HSD post-hoc results of DNA yield between each DNA extraction method. Significant p-values are in bold.

Method Pairs	Tukey HSD Q statistic	Tukey HSD p-value
M1 vs M2	6.2	**p** < **0**.**01**
M1 vs M3	6.87	**p** < **0**.**01**
M1 vs M4	9.22	**p** < **0**.**01**
M1 vs M5	9.37	**p** < **0**.**01**
M1 vs M6	10.46	**p** < **0**.**01**
M1 vs M7	13.38	**p** < **0**.**01**
M1 vs M8	12.68	**p** < **0**.**01**
M2 vs M3	0.68	0.9
M2 vs M4	3.02	0.41
M2 vs M5	3.17	0.35
M2 vs M6	4.27	0.08
M2 vs M7	6.76	**p** < **0**.**01**
M2 vs M8	6.49	**p** < **0**.**01**
M3 vs M4	2.34	0.69
M3 vs M5	2.5	0.63
M3 vs M6	3.59	0.21
M3 vs M7	6.04	**p** < **0**.**01**
M3 vs M8	5.81	**p** < **0**.**01**
M4 vs M5	0.15	0.9
M4 vs M6	1.25	0.9
M4 vs M7	3.53	0.22
M4 vs M8	3.47	0.24
M5 vs M6	1.09	0.9
M5 vs M7	3.37	0.28
M5 vs M8	3.31	0.3
M6 vs M7	2.2	0.75
M6 vs M8	2.22	0.74
M7 vs M8	0.17	0.9

**Figure 2:**
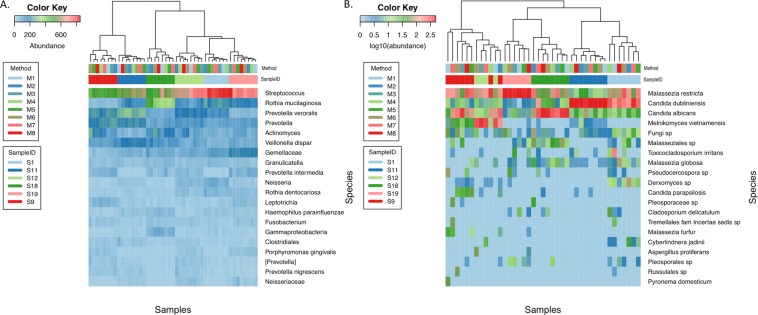
Heat Map of Bacterial and Fungal Species. (**A**) Bacterial heatmap. The top 20 bacterial OTUs for six oral samples processed by eight different extraction methods were used to construct a heatmap. OTUs were classified to species or lowest possible taxonomic level. Heatmap shows that samples cluster by patient (SampleID, 2^nd^ row), not extraction method (Method, 1^st^ row). (**B**) Fungal Heatmap. The top 20 fungal OTUs were used to construct a heatmap for the same samples described in panel A. Fungal OTUs were classified to species or lowest possible taxonomic level. Clustering demonstrated predominant grouping by individual (SampleID, 2^nd^ row) vs. method of extraction (Method, 1^st^ row). Legends to the left of the figures indicate color scheme for log transformed OTU abundance, method and sample in descending order.

DNA from the 48 DNA samples were amplified using 16 S rRNA V6 barcoded primers and recently described primers for the ITS1 region and submitted for Illumina NGS. Raw sequences were processed for quality control and chimera removal, resulting in a total of 373,840 bacterial reads (average of 7,788 ± 1,837 reads per sample), and 363,881 fungal sequence reads (average of 5,965 ± 1,579 reads per sample). The bacterial community composition and normalized abundances in the oral cavity are displayed in the heat map (Fig. 2A). Dendrogram clustering based on the top 20 species shows a tendency of samples to cluster by original subject. DNA extraction method did not show clustering. Community clustering based on the top 20 fungi (Fig. 2B) displays a closer distance between samples than seen with the bacterial 16 S data. However, the fungal heatmap also indicated that samples tended to cluster together based on subject and not extraction method.

Seven bacterial phyla were identified; *Firmicutes,* *Bacteroidetes*, *Actinobacteria*, *Fusobacteria*, *Proteobacteria*, *Spirochaetes*, and the candidate phylum *TM7* (also known as *Saccharibacteria*), with the majority of OTUs assigned to *Firmicutes* and *Bacteroidetes*. At the genus/species level, *Streptococcus* dominated the oral cavity, consistent with published studies^27^. *Rothia mucilaginosa*^34^, an opportunistic pathogen in immunocompromised patients and *Prevotella veroralis*, a biofilm forming opportunistic pathogen^35^, were the second and third most abundant species, respectively (Fig. 2A).

The oral mycobiota was dominated by species from *Ascomycota*, *Basidiomycota*, an unidentified fungal phyla, and *Zygomycota* (order based on cumulative dominance across all samples). Constituents of the *Candida* genus were amongst the top identified OTUs consistent with previous reports on the oral mycobiome^36^. Several species of *Malassezia* were also identified in the oral cavity, including *Malassezia restricta* (Fig. 2B), a common lipid dependent human pathogen that is usually found on skin^37^.

**Figure 3:**
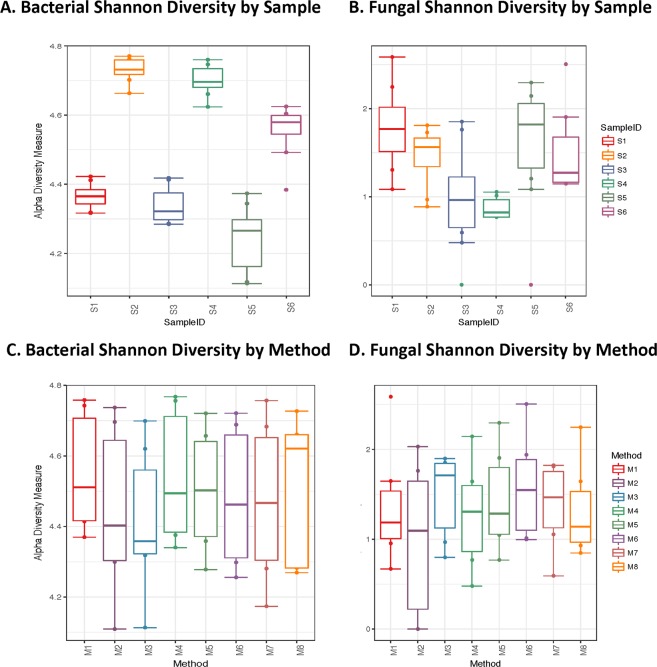
Comparison of Fungal and Bacterial Shannon Alpha Diversity Measures. Shannon alpha diversity box plots of bacterial and fungal community composition based on variance in species evenness is shown for samples (panels A and B) and by methods (panels C and D). Significant variance is observed in bacterial sample evenness, p < 0.001 (panel A) as well as fungal community evenness, p < 0.001 (panel B). No significant differences are observed for Shannon diversity based on collection method for bacterial, p = 0.87 (panel C) or fungal diversity measures, p = 0.93 (panel D).

**Figure 4:**
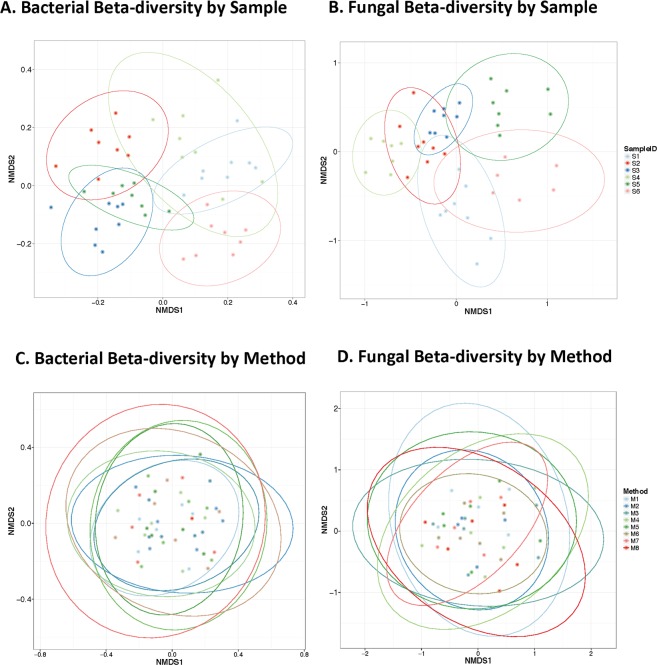
Beta-diversity Visualized Using Non-metric Multidimensional Scaling (NMDS) Plot With Bray-Curtis Dissimilarity Distances. NMDS plots on rank order Bray-Curtis distances were used to assess significance in bacterial and fungal community composition between individuals (panels A and B) and methods (panels C and D). Plot ellipses represent the 95% confidence regions for group clusters. Clustering by sample is highly significant for bacterial R^2^ = 0.80 p < 0.001 (panel A) and fungal communities R^2^ = 0.84 p < 0.001 (panel B) communities. DNA isolation method did not exhibit significant clustering in either bacterial R^2^ = 0.086 p = 0.996 (panel C) or fungal communities R^2^ = 0.039 p = 1.00 (panel D). Significance was determined using PERMANOVA analyses.

Significant variation in sample evenness, based on the Shannon diversity index, was observed in the bacterial p < 0.001 and fungal p < 0.001 assays (Fig. 3A,B respectively). There was no significant difference in the Shannon diversity index among DNA extraction methods for either the bacterial p = 0.87 or fungal assays p = 0.93 (Fig. 3C,D, respectively). Similarly, β-diversity showed distinct clusters formed on the basis of subject in both the bacterial p < 0.001 and fungal p < 0.001 community analyses, which explained nearly all of the inter-sample community variance, R^2^ = 0.80 and R^2^ = 0.84, respectively (Fig. 4A,B). β-diversity analyses did not show significant sample clustering based on extracted method for either bacteria R^2^ = 0.086, p = 0.996 or fungi R^2^ = 0.039, p = 1.00 (Fig. 4C,D, respectively).

## Discussion

In the current study, eight methods for DNA extraction from six oral cavity samples were used and DNA quantity and microbial community composition were compared. Our analysis revealed that DNA yield was significantly different among the eight DNA extraction methods with DNA recovery greatest after phenol-chloroform extraction (Fig. 1). The lower DNA yield of commercially available kits (Table 1) compared to the phenol-chloroform extraction method may be due to DNA loss during silica column purification. DNA yield tended to be greater with enzymatic digestion than using mechanical lysis (bead-beating) approaches. The lower DNA yield among bead-beaten samples is likely due to DNA degradation during mechanical disruption. Thus, for DNA yield, non-bead-beating methods, particularly phenol-chloroform extraction provides the greatest yield of total DNA.

Although DNA for human genetic studies has frequently been obtained using oral mouthwash and/or saliva collection methods^38^, compatibility of the DNA from these studies for future microbiome studies has not been examined in detail. Previous studies found differences in the oral bacterial microbiome based on DNA extraction methods^32,33,39^; whereas, other studies determined that DNA extraction methods did not seem to influence major differences in the oral microbiome^22,31,40^. Nevertheless, it is hard to do a direct comparison amongst studies in the literature, since many used saliva and/or plaque collection^31,33,39,40^, some compared crude DNA to purified DNA^39,40^, others used mock communities^39^, and one did not include NGS analysis of the microbiome^32^. Only one study examined both bacterial and fungal communities and surprisingly found no differences amongst 4 methods for bacterial communities, but found phenol-chloroform extraction best for fungal community diversity^33^.

Although we found that DNA extraction methods had an influence on DNA yield, we did not find a significant difference in oral microbiome composition across eight DNA extraction methods of oral rinse specimens. Shannon diversity measures for bacterial and fungal communities were similar across the employed extraction methods and did not achieve statistically significant differences. Similarly, PERMANOVA analysis on rank order Bray distances did not demonstrate differences in β-diversity for either assay. Our results instead demonstrated that individual subject differences drove diversity measures across both bacteria and fungi. Taken together, these data suggest that both α- and β-diversity measures were consistent for all eight-extraction measures, and that the choice of method does not have a major influence on the observed oral communities. The results of this study might have been influenced by the larger number of samples analyzed compared to previous studies.

All eight extraction methods were able to consistently recapitulate the original subject microbiotas as indicated by both alpha and beta diversity measures including Shannon diversity index and Bray-Curtis distances, respectively. These findings are consistent with previous studies that have demonstrated that each person’s oral microbiome is unique^41,42^. Additionally, all methods reported here detected hard to lyse gram-positive species, such as *Streptococcus*^43^, indicating sufficient lysis of cells. Moreover, the similarity of results for fungal community analyses across all methods is consistent with the one report that found phenol-chloroform extraction yielded the highest fungal diversity in saliva^33^.

In summary, our study compared eight DNA extraction methods tested on oral rinse specimens that are commonly collected in large epidemiological studies and are used or may be used in the future to study the oral microbiome. While the eight methods tested in this study had significantly different DNA recovery, there was no difference in the observed oral microbiotas among methods. This study provides empiric evidence that research studies can select an appropriate DNA extraction method with or without bead-beating for characterization of the oral microbiota without influencing differences between the oral microbiome/mycobiome of individuals.

## Materials and Methods

### Consent and Approval for Use of Human Participants

Oral rinse specimens from six individuals were collected as part of a pilot study on sampling procedures for the Health and Nutrition Examination Survey in New York City 2013 (NYC HANES 2013), a collaborative project between the City University of New York (CUNY) Graduate School of Public Health and Health Policy and the NYC Department of Health and Mental Hygiene. IRB approval for analysis of pilot oral specimens was obtained from the Human Research Protection Program (HRPP) of CUNY. All methods performed in this study were conducted in accordance with Hunter College (CUNY) university integrated IRB approved protocol (PT: 346358-9). Informed consent was obtained from study participants prior to sample collection. Upon receipt all used human specimens received a lab Sample ID and no information regarding, age, race, gender or any other identifying information was used in the presented study.

### Specimen Collection

Consented study participants provided an oral sample by rinsing with 20 mL of Scope mouthwash for 20 seconds. The 20-second oral rinse was broken into two 5-second swish sessions and two 5-second gargle sessions. The oral rinse samples were frozen at −80 °C at the New York State Public Health Laboratory (NYPHL) office and were transported on dry ice to Albert Einstein College of Medicine, where they were immediately stored at −80 °C.

### DNA Extraction

DNA was extracted from the oral rinse samples using eight DNA extraction methods based on physical and/or enzymatic lysis steps and isolation procedures (Table 1). Extraction methods with commercially available kits all used a silica-based column. One extraction method included a non-commercial method using phenol-chloroform. All DNA isolation methods evaluated in this study are either commonly used in DNA extraction or have previously been used in microbial analysis studies. For each method, 1 mL from each oral rinse sample was centrifuged (5,000 × g) for 5 minutes. The cell pellet was re-suspended in 100 μl TE buffer (10 mM Tris. Cl, pH 8.0, 1 mM EDTA) and used for DNA extraction. Upon completion of each extraction method, the purified DNA was eluted in 100 µl of elution buffer (pH 8.0) and DNA concentration was determined using a NanoDrop 2000 (Thermo Scientific, DE).

### Method 1 (Proteinase K/SDS/phenol chloroform extraction)

The cell pellet was directly processed in 200 μl cell lysis buffer (10 mmol/L Tris/HCl pH 8.0, 10 mmol/L EDTA, 0.1 mol/L NaCl, 2% SDS pH 8.0) and 10 μl proteinase K (20 mg/ml, Roche Diagnostics), and incubated overnight at 55 °C. The samples were treated with RNase A (100 mg/ml, Qiagen, Valencia, CA) for 20 minutes at 37 °C followed by phenol/chloroform extraction using Phase Lock Gel Tubes (PLG, 5 Prime Inc., Gaithersburg, MD) as described by the manufacturer.

### Method 2 (QIAamp DNA mini kit)

First, 20 μl of proteinase K (20 mg/ml) and 100 μl of Buffer AL were added to 100 μl of pelleted cells in TE. The samples were incubated at 56 °C for 10 minutes. After incubation, 100 μl of 100% ethanol was added to the samples and the DNA was purified following the manufacturer’s instructions.

### Method 3 (Enzymatic lysis followed by QIAamp DNA mini kit)

The pelleted cells in 100 µl TE were treated with lysozyme (0.84 mg/ml, Sigma Aldrich), mutanolysin (0.25 U/ml, Sigma Aldrich) and lysostaphin (21.10 U/ml, Sigma Aldrich) at 37 °C for 30 minutes. Subsequently, 20 μl proteinase K and 100 μl Buffer AL were added followed by incubation at 56 °C for 10 minutes. DNA was purified using the QIAamp DNA mini kit as described above.

### Method 4 (Enzymatic and bead-beating lysis followed by QIAamp DNA mini kit)

Pelleted cells were digested using enzymes as in Method 3. After incubation, the mixture was treated with 15 μl proteinase K (10 mg/ml) and 150 μl Buffer AL (Qiagen) at 56 °C for 10 minutes. The samples were then transferred to a clean screw-cap tube containing 300 mg of 0.1 mm-diameter zirconia/silica beads (BioSpec, Bartlesville, OK) and mechanically lysed using a FastPrep-24 Instrument (MP Biomedicals, Santa Ana, CA) at 6.0 m/s for 40 seconds. Next, the samples were centrifuged (10,000 × g) for 30 seconds and 200 μl of the supernatant was added to a clean microcentrifuge tube containing 100 μl of 100% ethanol. DNA was isolated using the QIAamp DNA Mini Kit (Qiagen) as described above.

### Method 5 (Enzymatic lysis followed by PowerLyzer PowerSoil DNA Isolation Kit)

The pelleted cells were incubated with the enzymes described in Method 3. After incubation, the mixture was transferred to a PowerLyzer Glass Bead Tube (0.1 mm) containing 650 μl of Bead Solution. The remainder of the DNA isolation protocol was continued beginning with step 4 of the PowerLyzer PowerSoil DNA Isolation Kit instructions (MO BIO laboratories, Inc., Carlsbad, CA). The bead-beating step used a FastPrep-24 Instrument (MP Biomedicals) set at 6.0 m/s for 40 seconds.

### Method 6 (PowerSoil DNA Isolation Kit)

DNA was extracted using the PowerSoil DNA Isolation Kit (MO BIO laboratories, Inc.) following the manufacturer’s protocol without additional enzymatic lysis. The cells were mechanically lysed using manufacturer’s provided bead tubes and a FastPrep-24 Instrument (MP Biomedicals) at 6.0 m/s for 40 seconds.

### Method 7 (UltraClean Microbial DNA Isolation Kit)

DNA was extracted using the UltraClean Microbial DNA Isolation Kit (MO BIO laboratories, Inc.) following the manufacturer’s protocol. The cells were mechanically lysed using manufacturer’s provided bead tubes and a FastPrep-24 Instrument (MP Biomedicals) at 6.0 m/s for 40 seconds.

### Method 8 (BiOstic Bacteremia DNA Isolation Kit)

DNA was isolated using the BiOstic Bacteremia DNA Isolation Kit (MO BIO laboratories, Inc.) following the manufacturer’s protocol. The cells were mechanically lysed using manufacturer’s provided bead tubes and a FastPrep-24 Instrument (MP Biomedicals) at 6.0 m/s for 40 seconds.

### 16S rRNA gene and ITS1 region amplification and massively parallel sequencing

To amplify the 16SrRNA gene region of bacterial species, an aliquot of 0.5 µl DNA from each sample and DNA isolation method was PCR amplified in a total reaction volume of 25 µl using barcoded primers spanning the V6 variable region of the 16 S rRNA gene as previously described^26^. In brief, an equal mixture of AmpliTaq Gold (Applied Biosystems, Carlsbad, CA) and HotStart-IT FideliTaq DNA Polymerase (Affymetrix, Santa Clara, CA) was used. For all samples a unique 8-bp barcode was introduced to the PCR amplicons on the primers. Thermocycling conditions included an initial denaturation at 95 °C for 5 minutes, then 15 cycles at 95 °C for 1 minute, 55 °C for 1 minute, and 68 °C for 1 minute. This was followed by 15 cycles at 95 °C for 1 minute, 60 °C for 1 minute, and 68 °C for 1 minute; and a final extension for 10 minutes at 68 °C.

To amplify the ITS1 region of fungal species, 10 µl from each sample and DNA isolation method was PCR amplified in a total reaction volume of 25 µl using barcoded primers specific to the ITS1 region of the fungal ribosomal gene cluster^44^. In brief, 9.25 µl of dd H_2_O, 2.5 µl of USB 10X buffer with MgCl_2_ (10 mM Tris-HCl, pH 8.6, 50 mM KCl, 1.5 Mm MgCl_2_, Affymetrix, Santa Clara, CA), 1 µl of USB MgCl2 (25 mM), 0.5 µl of dNTP mix (10 mM each, Roche Basel, Switzerland), 0.25 µl AmpliTaq Gold, polymerase (5 U/µl, Applied Biosystems, Carlsbad, CA), 0.5 µl of HotStart-IT DNA FideliTaq Polymerase (2.5 U/µl, Affymetrix), and 1 µl (5 µM) of each primer (IDT, Coralville, IA). Thermocycling included an initial denaturation of 95 °C for 3 mins, followed by 35 cycles of 95 °C for 30 s, 55 °C for 30 s, 68 °C for 2 min, followed by a final extension of 68 °C for 10 min.

The 16 S rRNA and ITS1 PCR products each were pooled at approximately equal molar DNA concentrations and purified using the QIAquick Gel Extraction Kit (Qiagen). Following library preparation using TruSeq DNA Sample Prep Kits (Illumina, San Diego, CA), the pooled 16SrRNA DNA was sequenced on an Illumina HiSeq. 2500 using paired-end 150 bp reads, while the pooled ITS1 DNA was sequenced on an Illumina MiSeq using paired-end 300 bp reads, by the Epigenomics and Genomics Core Facility, Albert Einstein College of Medicine (Bronx, NY).

### Bioinformatics

MiSeq reads were demultiplexed using novocraft’s novobarcode 1.00^45^ based on sample specific barcodes^46^. Reads were left and right trimmed with PrinSeq. 0.20.4^47^ to remove bases that fell below the PHRED score of 25. Paired end reads were merged with PANDASEQ. 1.20^48^ using default settings.

For 16S rRNA gene reads, OTUs were clustered using closed reference selection with USEARCH using a custom in-house database that contains reference sequences from Green-Genes 13.8^49^. Additionally reference sequences of an oral microbiome specific database, Human Oral Microbiome Database (HOMD)^50^, were retrieved in order to account for bacteria specific to the human oral cavity. Representative sequences were aligned using PyNAST^51^ and phylogenic analyses were performed using FastTree 2.0^52^.

For fungal ITS1 reads, open reference OTU picking was employed with QIIME 1.9^53^ open-reference OTU picking protocol as previously described^44^. The protocol was modified to use VSEARCH version 1.4.0^54^, which allowed for higher throughput. The OTU clustering threshold was changed from 97% to 99% sequence identity to account for fungal diversity. Sequence dereplication and chimera removal was performed as part of the QIIME’s usearch quality control protocol prior to OTU picking with VSEARCH. Representative sequences for each OTU cluster were chosen based on sequence abundance. BLAST was used to assign the taxonomy^55^.

All data were processed in R version 3.2.1^56^. QIIME outputs were imported into R using the *phyloseq*^57^. package and further processed with *vegan*^58^, *coin*^59,60^, and *reshape2*^60^. Data visualization was performed using *ggplot2*^61^. General community clustering was performed on the 20 most abundant OTUs (in terms of mean abundance across all samples) collapsed based on shared taxonomy at the species level using ward.D2 hierarchical clustering. β-diversity was assessed using Bray-Curtis distances and significance was calculated with PERMANOVA using the adonis function from the *vegan* package^58^. Statistical ellipses from the *ggplot2* package were used to visualize the sample and method clusters on the NMDS plots. α-diversity was analyzed based on the Shannon’s alpha diversity and observed number of OTUs metrics and significance was determined using the Kruskal-Wallis test.

## Data Availability

Data used in current study is available from the corresponding author upon reasonable request.

## References

[1] Benn AM, Heng NC, Broadbent JM, Thomson WM (2018). Studying the human oral microbiome: challenges and the evolution of solutions. Australian dental journal.

[2] Yamashita Y, Takeshita T (2017). The oral microbiome and human health. Journal of oral science.

[3] Takahashi N, Nyvad B (2011). The role of bacteria in the caries process: ecological perspectives. J Dent Res.

[4] Liu, B. *et al*. Deep Sequencing of the Oral Microbiome Reveals Signatures of Periodontal Disease. *PLoS One* **7** (2012). 10.1371/journal.pone.0037919 PMC3366996 22675498

[5] Coventry J, Griffiths G, Scully C, Tonetti M (2000). Periodontal disease. BMJ.

[6] Kuo LC, Polson AM, Kang T (2008). Associations between periodontal diseases and systemic diseases: a review of the inter-relationships and interactions with diabetes, respiratory diseases, cardiovascular diseases and osteoporosis. Public Health.

[7] Meurman JH, Sanz M, Janket SJ (2004). Oral health, atherosclerosis, and cardiovascular disease. Crit Rev Oral Biol Med.

[8] Huang YJ, Lynch SV (2011). The emerging relationship between the airway microbiota and chronic respiratory disease: clinical implications. Expert Rev Respir Med.

[9] de Pablo P, Chapple IL, Buckley CD, Dietrich T (2009). Periodontitis in systemic rheumatic diseases. Nat Rev Rheumatol.

[10] Tezal M (2009). Chronic periodontitis and the incidence of head and neck squamous cell carcinoma. Cancer Epidemiol Biomarkers Prev.

[11] Michaud DS, Liu Y, Meyer M, Giovannucci E, Joshipura K (2008). Periodontal Disease, Tooth Loss and Cancer Risk in a Prospective Study of Male Health Professionals. Lancet Oncol.

[12] Saini R, Saini S, Saini SR (2010). Periodontitis: A risk for delivery of premature labor and low-birth-weight infants. J Nat Sci Biol Med.

[13] Hayes, R. B. *et al*. Association of Oral Microbiome With Risk for Incident Head and Neck Squamous Cell Cancer. *JAMA oncology*, 10.1001/jamaoncol.2017.4777 (2018). 10.1001/jamaoncol.2017.4777 PMC5885828 29327043

[14] Whitmore SE, Lamont RJ (2014). Oral bacteria and cancer. PLoS pathogens.

[15] Lamont, R. J., Koo, H. & Hajishengallis, G. The oral microbiota: dynamic communities and host interactions. *Nature Reviews Microbiology*, 1 (2018). 10.1038/s41579-018-0089-x PMC6278837 30301974

[16] Mukherjee PK (2018). Dysbiosis in the oral bacterial and fungal microbiome of HIV-infected subjects is associated with clinical and immunologic variables of HIV infection. PloS one.

[17] Klimesova K, Jiraskova Zakostelska Z, Tlaskalova-Hogenova H (2018). Oral bacterial and fungal microbiome impacts colorectal carcinogenesis. Frontiers in microbiology.

[18] Delaney, C. *et al*. Fungi at the Scene of the Crime: Innocent Bystanders or Accomplices in Oral Infections? *Current Clinical Microbiology Reports*, 1-11 (2018).

[19] Paster BJ (2001). Bacterial Diversity in Human Subgingival Plaque. J Bacteriol.

[20] Consortium HMP (2012). A framework for human microbiome research. Nature.

[21] von Wintzingerode F, Gobel UB, Stackebrandt E (1997). Determination of microbial diversity in environmental samples: pitfalls of PCR-based rRNA analysis. FEMS Microbiol Rev.

[22] Wu J, Lin I-H, Hayes RB, Ahn J (2014). Comparison of DNA extraction methods for human oral microbiome research. British Journal of Medicine and Medical Research.

[23] Feigelson HS (2001). Determinants of DNA yield and quality from buccal cell samples collected with mouthwash. Cancer Epidemiol Biomarkers Prev.

[24] Garcia-Closas M (2001). Collection of genomic DNA from adults in epidemiological studies by buccal cytobrush and mouthwash. Cancer Epidemiol Biomarkers Prev.

[25] Lum A, Le Marchand L (1998). A simple mouthwash method for obtaining genomic DNA in molecular epidemiological studies. Cancer Epidemiol Biomarkers Prev.

[26] Smith BC (2012). The cervical microbiome over 7 years and a comparison of methodologies for its characterization. PloS one.

[27] Aas JA, Paster BJ, Stokes LN, Olsen I, Dewhirst FE (2005). Defining the normal bacterial flora of the oral cavity. J Clin Microbiol.

[28] Dewhirst FE (2010). The human oral microbiome. Journal of bacteriology.

[29] Eren AM, Borisy GG, Huse SM, Mark Welch JL (2014). Oligotyping analysis of the human oral microbiome. Proc Natl Acad Sci USA.

[30] Melka, R., Fnu, N., Morales, J. F. & Loewy, Z. G. Pharmacy: Oral Microbiome: Relationship of Stomatitis of Denture Wearers and Chronic Obstructive Pulmonary Disease (COPD) (2018).

[31] Lim Y, Totsika M, Morrison M, Punyadeera C (2017). The saliva microbiome profiles are minimally affected by collection method or DNA extraction protocols. Scientific reports.

[32] Sohrabi M (2016). The yield and quality of cellular and bacterial DNA extracts from human oral rinse samples are variably affected by the cell lysis methodology. Journal of microbiological methods.

[33] Vesty A, Biswas K, Taylor MW, Gear K, Douglas RG (2017). Evaluating the Impact of DNA Extraction Method on the Representation of Human Oral Bacterial and Fungal Communities. PloS one.

[34] Cho EJ, Sung H, Park SJ, Kim MN, Lee SO (2013). Rothia mucilaginosa Pneumonia Diagnosed by Quantitative Cultures and Intracellular Organisms of Bronchoalveolar Lavage in a Lymphoma Patient. Ann Lab Med.

[35] Binkley CJ, Haugh GS, Kitchens DH, Wallace DL, Sessler DI (2009). Oral microbial and respiratory status of persons with mental retardation/intellectual and developmental disability: an observational cohort study. Oral Surg Oral Med Oral Pathol Oral Radiol Endod.

[36] Ghannoum MA (2010). Characterization of the oral fungal microbiome (mycobiome) in healthy individuals. PLoS pathogens.

[37] Velegraki A, Cafarchia C, Gaitanis G, Iatta R, Boekhout T (2015). Malassezia infections in humans and animals: pathophysiology, detection, and treatment. PLoS pathogens.

[38] Agalliu I (2016). Associations of Oral alpha-, beta-, and gamma-Human Papillomavirus Types With Risk of Incident Head and Neck Cancer. JAMA oncology.

[39] Abusleme, L., Hong, B. Y., Dupuy, A. K., Strausbaugh, L. D. & Diaz, P. I. Influence of DNA extraction on oral microbial profiles obtained via 16S rRNA gene sequencing. *Journal of oral microbiology* **6**, 10.3402/jom.v6.23990 (2014). 10.3402/jom.v6.23990 PMC4000428 24778776

[40] Lazarevic V, Gaia N, Girard M, Francois P, Schrenzel J (2013). Comparison of DNA extraction methods in analysis of salivary bacterial communities. PloS one.

[41] Langmead B, Salzberg SL (2012). Fast gapped-read alignment with Bowtie 2. Nature methods.

[42] Ding T, Schloss PD (2014). Dynamics and associations of microbial community types across the human body. Nature.

[43] Biesbroek, G. *et al*. Deep Sequencing Analyses of Low Density Microbial Communities: Working at the Boundary of Accurate Microbiota Detection. *PLoS One* **7** (2012). 10.1371/journal.pone.0032942 PMC3295791 22412957

[44] Usyk M, Zolnik CP, Patel H, Levi MH, Burk RD (2017). Novel ITS1 Fungal Primers for Characterization of the Mycobiome. mSphere.

[45] Hercus, C. Novocraft short read alignment package. http://www.novocraft.com (2009).

[46] Hamady M, Walker JJ, Harris JK, Gold NJ, Knight R (2008). Error-correcting barcoded primers for pyrosequencing hundreds of samples in multiplex. Nature methods.

[47] Schmieder R, Edwards R (2011). Quality control and preprocessing of metagenomic datasets. Bioinformatics.

[48] Masella AP, Bartram AK, Truszkowski JM, Brown DG, Neufeld JD (2012). PANDAseq: paired-end assembler for illumina sequences. BMC bioinformatics.

[49] DeSantis TZ (2006). Greengenes, a chimera-checked 16S rRNA gene database and workbench compatible with ARB. Applied and environmental microbiology.

[50] Chen, T. *et al*. The Human Oral Microbiome Database: a web accessible resource for investigating oral microbe taxonomic and genomic information. *Database* **2010** (2010). 10.1093/database/baq013 PMC2911848 20624719

[51] Caporaso JG (2009). PyNAST: a flexible tool for aligning sequences to a template alignment. Bioinformatics.

[52] Price MN, Dehal PS, Arkin AP (2010). FastTree 2–approximately maximum-likelihood trees for large alignments. PloS one.

[53] Caporaso JG (2010). QIIME allows analysis of high-throughput community sequencing data. Nature methods.

[54] Rognes T, Flouri T, Nichols B, Quince C, Mahé F (2016). VSEARCH: a versatile open source tool for metagenomics. PeerJ.

[55] Altschul SF, Gish W, Miller W, Myers EW, Lipman DJ (1990). Basic local alignment search tool. Journal of molecular biology.

[56] R: A language and environment for statistical computing (R Foundation for Statistical Computing, Vienna, Austria 2014).

[57] McMurdie PJ, Holmes S (2013). phyloseq: an R package for reproducible interactive analysis and graphics of microbiome census data. PloS one.

[58] Oksanen J (2007). The vegan package. Community ecology package.

[59] Batdorf, C. S. (Google Patents, 1903).

[60] Wickham, H. reshape2: Flexibly reshape data: a reboot of the reshape package. *R package version* **1** (2012).

[61] Wickham, H. *ggplot2: elegant graphics for data analysis*. (Springer, 2016).

